# Duration of patients’ visits to the hospital emergency department

**DOI:** 10.1186/1471-227X-12-15

**Published:** 2012-11-06

**Authors:** Zeynal Karaca, Herbert S Wong, Ryan L Mutter

**Affiliations:** 1 Social & Scientific Systems, Inc.; George Washington University, Health Policy Department; Agency for Healthcare Research and Quality, Center for Delivery, Organization and Markets, 540 Gaither Road, Rockville, MD, 20850, USA; 2 Agency for Healthcare Research and Quality, Agency for Healthcare Research and Quality, Center for Delivery, Organization and Markets, 540 Gaither Road, Rockville, MD, 20850, USA

## Abstract

**Background:**

Length of stay is an important indicator of quality of care in Emergency Departments (ED). This study explores the duration of patients’ visits to the ED for which they are treated and released (T&R).

**Methods:**

Retrospective data analysis and multivariate regression analysis were conducted to investigate the duration of T&R ED visits. Duration for each visit was computed by taking the difference between admission and discharge times. The Healthcare Cost and Utilization Project (HCUP) State Emergency Department Databases (SEDD) for 2008 were used in the analysis.

**Results:**

The mean duration of T&R ED visit was 195.7 minutes. The average duration of ED visits increased from 8 a.m. until noon, then decreased until midnight at which we observed an approximately 70-minute spike in average duration. We found a substantial difference in mean duration of ED visits (over 90 minutes) between Mondays and other weekdays during the transition time from the evening of the day before to the early morning hours. Black / African American patients had a 21.4-minute longer mean duration of visits compared to white patients. The mean duration of visits at teaching hospitals was substantially longer than at non-teaching hospitals (243.8 versus 175.6 minutes). Hospitals with large bed size were associated with longer duration of visits (222.2 minutes) when compared to hospitals with small bed size (172.4 minutes) or those with medium bed size (166.5 minutes). The risk-adjusted results show that mean duration of visits on Mondays are longer by about 4 and 9 percents when compared to mean duration of visits on non-Monday workdays and weekends, respectively.

**Conclusions:**

The duration of T&R ED visits varied significantly by admission hour, day of the week, patient volume, patient characteristics, hospital characteristics and area characteristics.

## Background

Length of stay (LOS) is perceived as an important indicator of quality of care in Emergency Departments (EDs) [1]. Increased LOS at EDs may contribute to systematic problems in the delivery of efficient and high quality medical care in the U.S [2]. Increased LOS may mean that patients wait longer to see ED physicians and to obtain critical treatments and test results [3]. Among the thoughtful measures related to duration in the ED that are of interest to policymakers and providers are door to diagnostic time, door to treatment time (including the provision of pain medicine for certain conditions), ED arrival to ED departure time, and decision to admit to ED departure time for patients that are admitted. The Centers for Medicare & Medicaid Services (CMS) began data collection on three ED throughput timing measures on January 1, 2012.

There is a growing body of literature on the factors associated with longer ED LOS. Researchers deconstructed the association between static crowding measures (waiting room volume, census, number boarding, and inpatient occupancy) and waiting room, treatment, and boarding times experienced by ED patients [4]. The literature finds that when more people are waiting to be treated, intervals between phases of care at EDs lengthen and the waiting line becomes longer. This also illustrates the fundamental relationship between crowding (waiting lines) and delays in patient care [5]. ED LOS is positively associated with the hospital occupancy rate and number of emergency admissions [6]. The crowding factors increase waiting and boarding time but not treatment time [7]. Increasing numbers of low-complexity patients do not significantly lengthen the waiting time or ED LOS for higher complexity patients [8]. Certain census variables (e.g., the number of admissions from the ED per day) and the number of intensive care and cardiac telemetry units affect ED length of stay across many hospital settings [9].

Increased LOS at EDs may contribute to ED crowding, which has become a major public health problem in the United States. ED crowding can contribute to poorer patient outcomes and to lost-demand for ED services (and the associated revenue) when patients leave without being seen [10]. ED crowding presents obvious operational and logistic problems for hospitals, and raises serious ethical concerns [11,12]. The moral problems posed by ED boarding and resultant crowding have a variety of undesirable consequences such as increased patient waiting times, decreased ability to protect patient privacy and confidentiality, impaired evaluation and treatment, and difficulties in delivering person-centered care [13].

This study uses a previously unused data source that captures ED visits for entire states to explore ED LOS by admission hour, day of the week, patient volume, patient characteristics, hospital characteristics, and area characteristics. ED visits are limited to those in which the patients are treated and released (T&R), i.e., not admitted to the same hospital. The study contributes to the existing literature in the following important way: Existing studies examining emergency department LOS, crowding, and resource use generally employ data drawn from a sample of ED visits, obtained from a survey, or tracked as part of a before-after intervention study [13]. One of the largest of these data files^a^ is a nationally representative sample of 138,569 ED visits over a 5-year period [2]. In contrast, our data file includes 4.9 million ED visits in a single year. Healthcare policies designed to provide solutions to increased ED LOS, ED crowding, and related issues may produce better outcomes when they are based on large databases. Such large databases may shed light on the wide variations in utilization patterns of ED services and the significant differences in patient-related and market-specific factors [14]. Our findings may inform public and private policymakers on a broad range of issues including, but not limited to, Monday volume, impact of hospital bed size and hospital status on the average duration of T&R ED visits, and differences in duration by race.

## Methods

### Study design and population

We conducted a retrospective data analysis to investigate the duration of ED visits using the Healthcare Cost and Utilization Project (HCUP)^b^ State Emergency Department Databases (SEDD) for 2008. HCUP is maintained by the Agency for Healthcare Research and Quality (AHRQ). HCUP databases are publicly available for all researchers and can be purchased through the HCUP Central Distributor.^c^ The SEDD employed in this study include data on 4.9 million T&R ED visits in three states: Arizona, Massachusetts, and Utah. In general, the SEDD provide detailed diagnoses and procedures, total charges, and patient demographics. Demographics include gender, age, race, and expected payment source (e.g., Medicare, Medicaid, private insurance, other insurance, and self-pay). However, the SEDD from these three states also provide admission and discharge time for 99.1 percent of all visits, from which duration^d^ may be calculated.

We obtained information about hospital characteristics (e.g., urban versus rural, ownership type, teaching status, bed size, and system member) from the 2008 American Hospital Association (AHA) Annual Survey Database and linked them to SEDD files using hospital identifiers. In addition, we obtained information about the trauma level of the hospital using the Trauma Information Exchange Program database (TIEP), collected by the American Trauma Society and the Johns Hopkins Center for Injury Research and Policy. Finally, we used the 2008 Area Resource File (ARF)^e^ to obtain county-level income information.

The proper measures of ED LOS and ED crowding are not straightforward [15]. Few investigators have attempted to develop models characterizing the completion times of different phases of emergency care. Multivariate linear regression techniques used to estimate ED waiting room time, treatment time, and boarding time for patients who were admitted or discharged from a hospital’s main ED or urgent care area [7]. Similarly, discrete-time survival analysis is applied to evaluate the effect of crowding on the different phases of ED care [4]. Both studies estimated the influence of various patient, temporal, and system factors on the mean or median completion times for different phases of emergency care. Few researchers [16] contributed to this literature by demonstrating that the degree of crowding in a hospital can be accurately measured.

Because the proper measures of ED LOS were not readily available in our data, we computed the duration for each visit by taking the difference between admission and discharge times, which is the total of the time patients waited in ED rooms plus their treatment time. Ideally, one would separate the times into components identified as important in the literature. Unfortunately, HCUP data lacks sufficient detail to do this.

### Statistical Analyses

We initially performed extensive secondary data analyses to explore ED LOS by admission hour, day of the week, patient volume, patient characteristics, hospital characteristics and area characteristics. The frequencies, means, medians, and 95% confidence intervals for several of these variables were based on data for all T&R ED visits (excluding encounters where there was evidence that the patient also received observation services) in Arizona, Massachusetts, and Utah during 2008. Duration was expressed in minutes measured as the difference between admission time and discharge time.

The mean (median) duration for a specific admission hour was measured as the mean (median) value of the durations of all visits admitted to EDs at that specific hour during 2008. The total volume of visits for a specific admission hour was measured as the total number of T&R visits to the EDs observed at that specific hour during 2008. (Note that it was not possible to include ED visits that resulted in subsequent admission to the hospital in the analysis.) We applied a similar approach when reporting the mean duration of ED visits across patient demographics and hospital characteristics. For example, the mean duration of ED visits for Medicare patients was measured as the total duration of T&R ED visits by all Medicare patients divided by the total number of T&R visits by Medicare patients during 2008. Data were analyzed with SAS 9.02 and Stata 12.

Severity of illness is an important factor that can affect the mean duration of ED visits. To further explore the potential relationship between the mean duration of visits and various disease groups, we grouped ED visits into major disease categories based on Clinical Classification Software—a diagnosis and procedure categorization scheme based on the International Classification of Diseases, 9th Revision, Clinical Modification (ICD-9-CM). While the HCUP SEDD provide all diagnosis codes for every visit, they may not clearly differentiate between the primary diagnosis codes and other diagnosis codes. Therefore, we used all diagnosis codes reported for each visit when developing our major disease categories.

While this study is mostly observational, we also investigated the factors affecting the duration of T&R ED visits using several multivariable regression models. We attempted to explain the variability in the duration of T&R ED visits using admission day of the week, admission hour of the day, and patient and hospital characteristics. More specifically, we estimated several regression models to examine factors associated with the duration of patients’ T&R ED visits. We initially estimated a linear regression model that controls for 1) admission day of the week; 2) patient characteristics including age, sex, race, primary payers, and major disease categories; and 3) hospital characteristics including hospital teaching status, hospital ownership status, trauma hospitals, hospital location, and hospital bed size. Next, we estimated the same model by further controlling for patients’ admission hour of the day. Then, we developed a third model based on the second model by incorporating hospital-specific dummy variables to increase the robustness of our results.

Several previous studies [17-20] showed that linear regression models that contain a response variable at the individual level and predictors at both individual and higher levels of analysis disregard correlation structures in the data emanating from common influences operating within groups. For example, hospital attributes such as teaching status, bed size, or location may impose distinct effects on the duration of patients’ visits to the EDs. Such “intra-class correlation” violates classical linear regression assumptions concerning random error, independence, and common variance. Mainly, in nested data containing two levels, the random error is composed of both individual and group-level components, and thus, independence of observations fails as a portion of the random error is attributable to the group error which is constant within each group. Ignoring this correlation may lead to underestimates of standard errors of coefficients and therefore overestimates of the significance levels of parameters in linear regression models. By nesting patients within hospitals, we estimated our fourth model, which is a random intercept two level model with level-1 predictors. This model allows intercepts to vary, and hence, duration of ED visits for each patient are predicted by the intercept that varies across hospitals. This model also provides information about intra-class correlations, which enable us to determine what fraction of variance in duration of patients’ visits to the EDs are due to patient characteristics and which are due to hospital characteristics. Following the approach in the previous studies [17,18], we used hospital means to centralize all variables pertinent to patient demographics. We also aimed to partition the variation in duration of patients’ visits to the EDs between patient and hospital level, which in turn provides us an intra-class correlation.

## Results

### Descriptive results

#### Admission hour and day of the week

**Figure 1:**
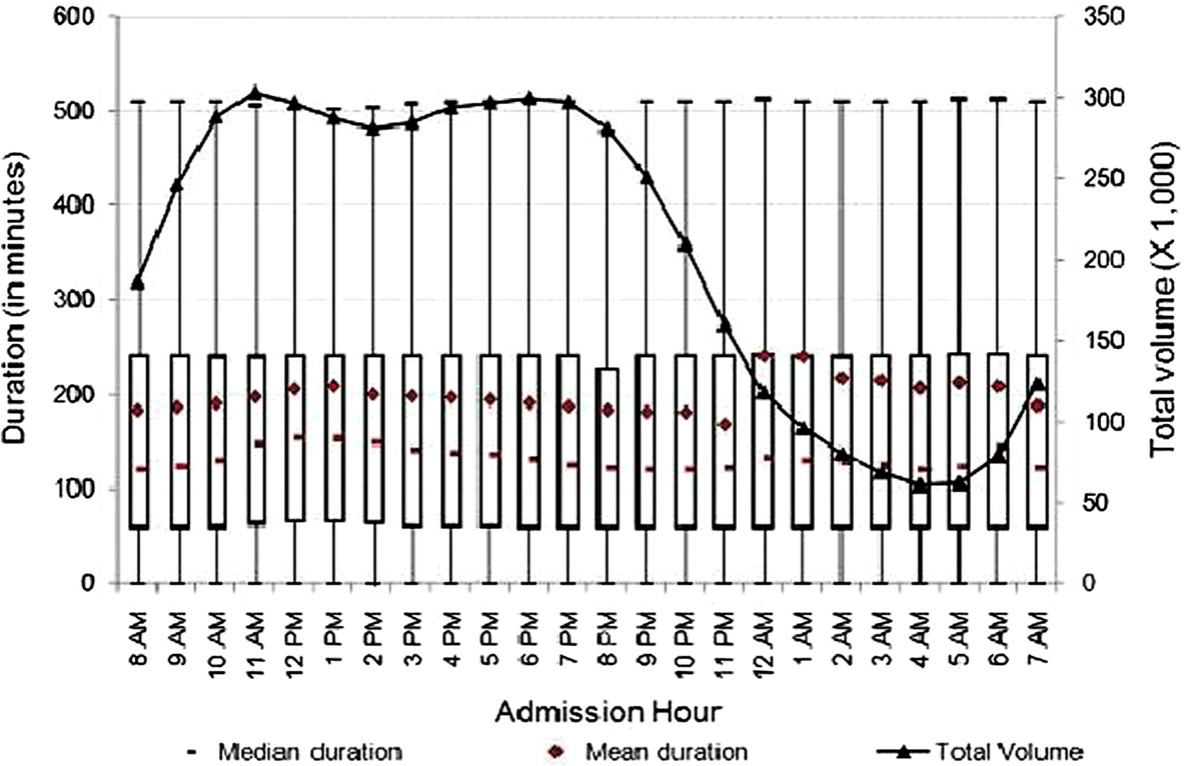
**Duration of treat-and-release visits at emergency departments by hour.** Data includes all treat-and-release emergency visits during 2008 in Arizona, Massachusetts and Utah. Duration is measured in minutes as the difference between admission time and discharge time for each visit.

Duration of visits varied substantially by admission hour and day of the week. At the 95th percentile, the mean duration of T&R ED visits was between 194.2 and 197.2 minutes. We found that the distribution of duration of ED visits was right-skewed. Therefore, we explored the relationship between total volume of visits with both mean and median duration at EDs by admission hour.^f^ As shown in Figure 1, the mean duration of ED visits increased from 8 a.m. until noon, then decreased until midnight at which time we observed an approximately 70-minute spike in mean duration. One plausible explanation for this might be that healthcare personnel change shifts at this time and/or a reduction in other resources between 11 p.m. and midnight. Another plausible explanation might be that healthcare personnel might experience a decrease in their labor productivity towards the end of their shifts. After midnight, we noticed decreases in duration of ED visits until early morning, and increases thereafter.

**Figure 2:**
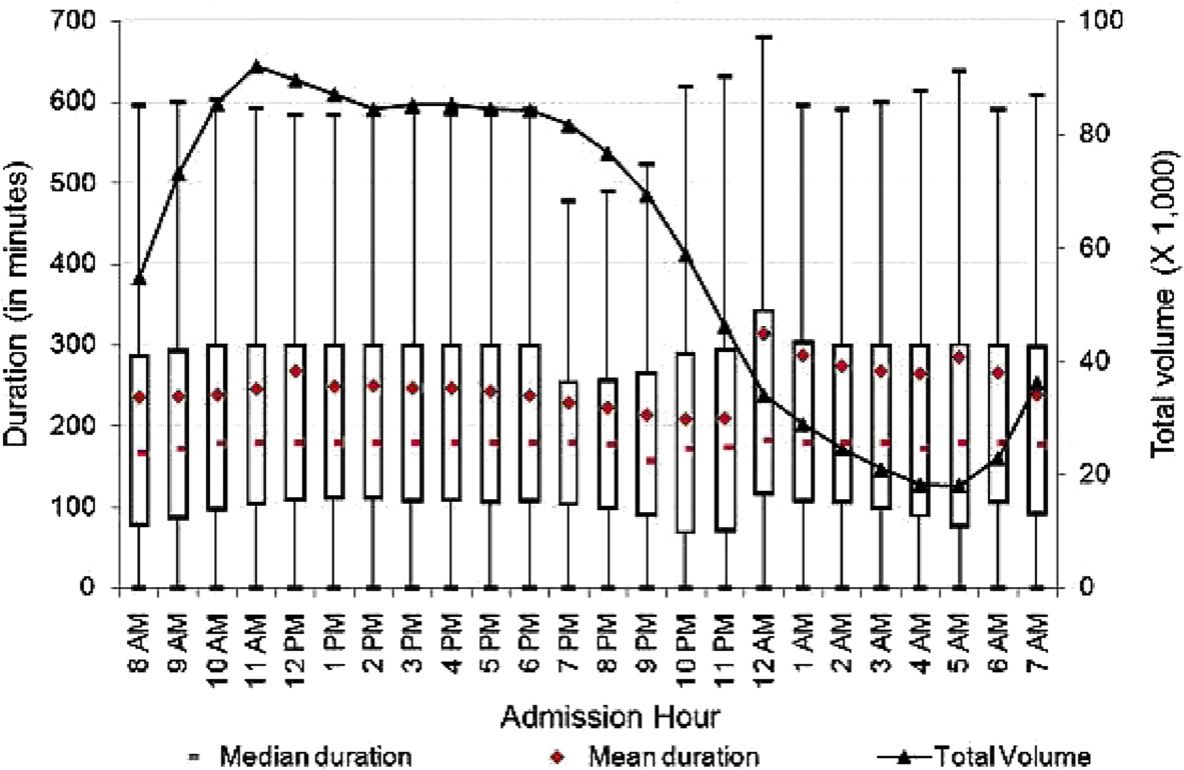
**Duration of treat-and-release visits at emergency departments of teaching hospitals by hour.** Data includes all treat-and-release emergency visits during 2008 in Arizona, Massachusetts and Utah. Duration is measured in minutes as the difference between admission time and discharge time for each visit.

**Figure 3:**
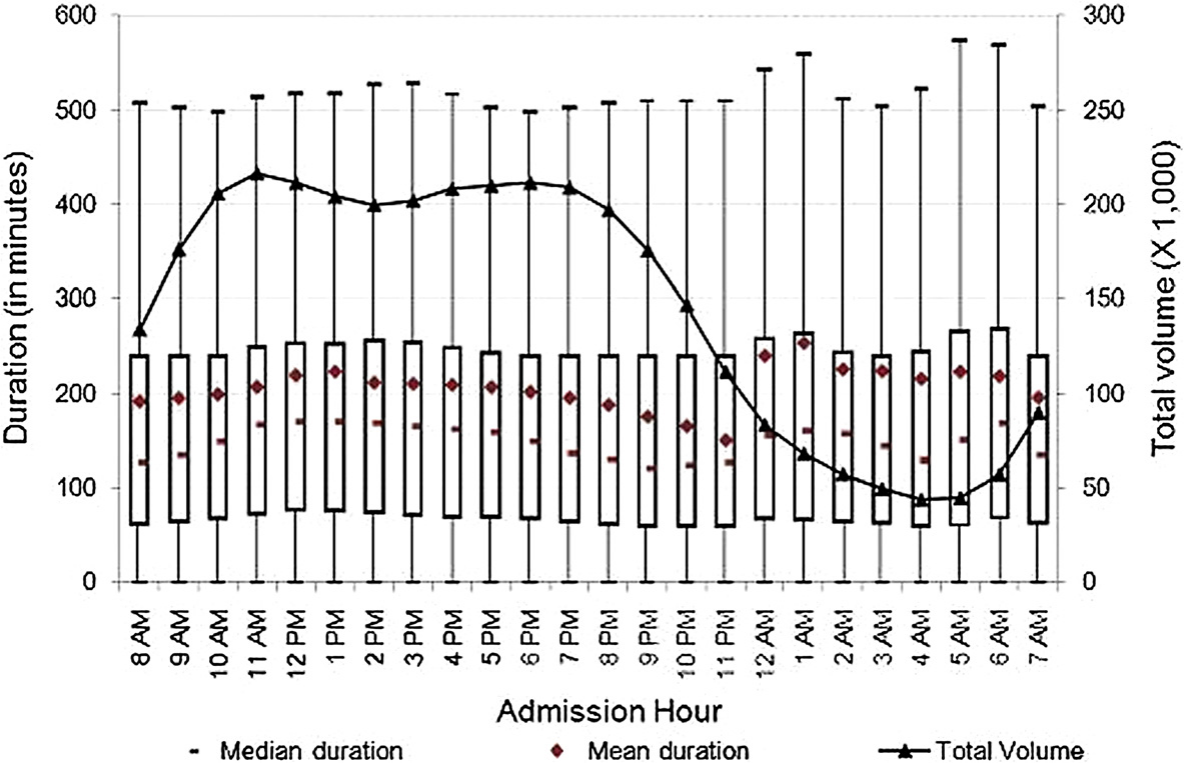
**Duration of treat-and-release visits at emergency departments of non-profit hospitals by hour.** Data includes all treat-and-release emergency visits during 2008 in Arizona, Massachusetts and Utah. Duration is measured in minutes as the difference between admission time and discharge time for each visit.

**Figure 4:**
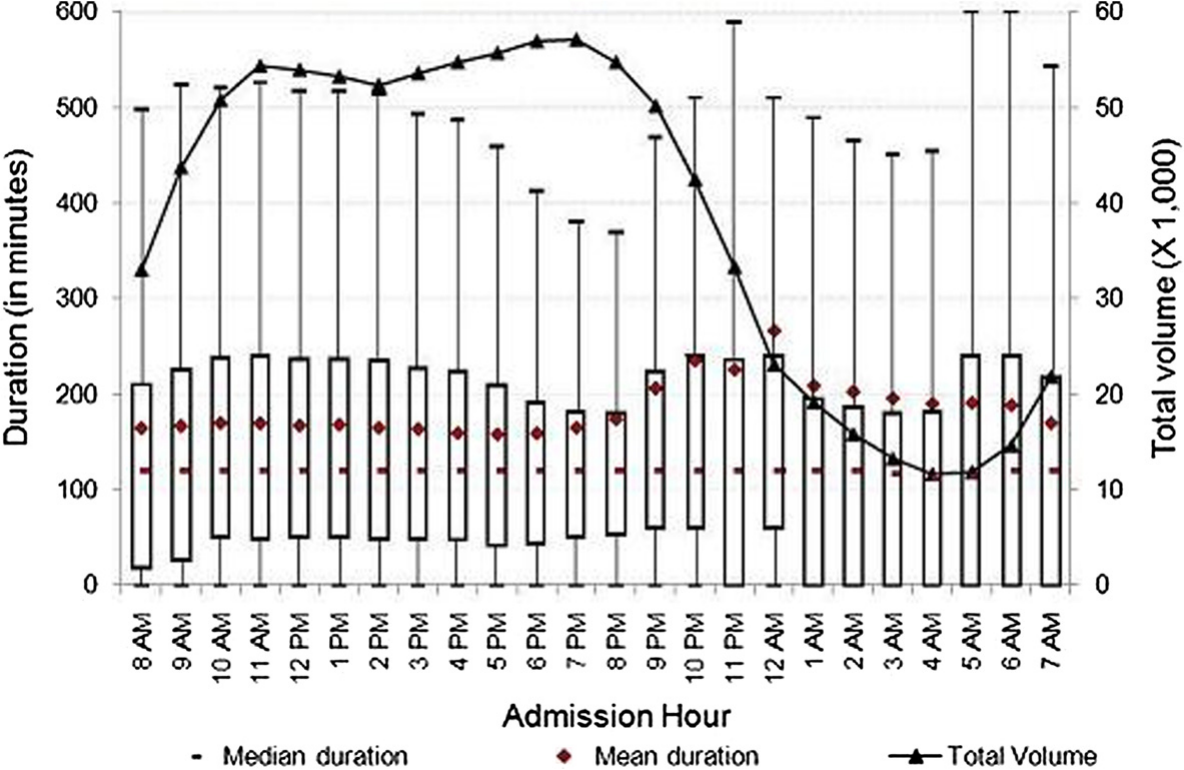
**Duration of treat-and-release visits at emergency departments of for-profit hospitals by hour.** Data includes all treat-and-release emergency visits during 2008 in Arizona, Massachusetts and Utah. Duration is measured in minutes as the difference between admission time and discharge time for each visit.

**Figure 5:**
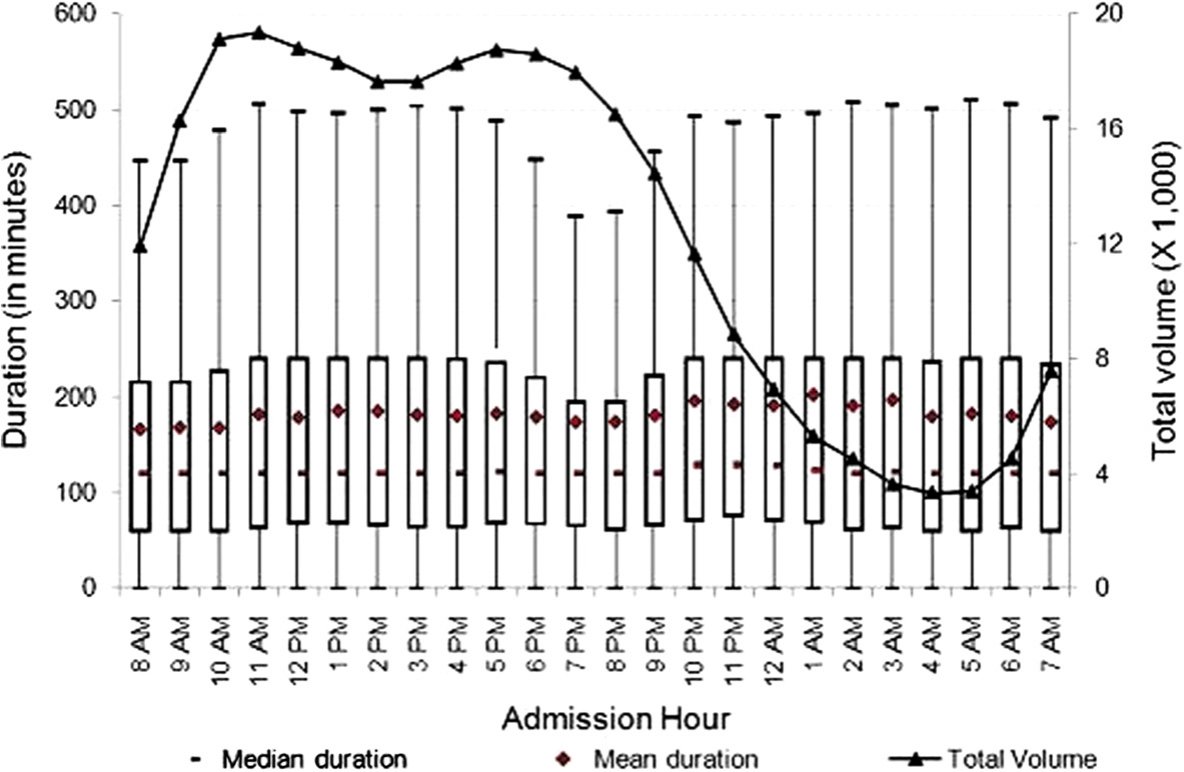
**Duration of treat-and-release visits at emergency departments of public hospitals by hour.** Data includes all treat-and-release emergency visits during 2008 in Arizona, Massachusetts and Utah. Duration is measured in minutes as the difference between admission time and discharge time for each visit.

Next, we explored the relationship between total number of visits and admission hour. As presented in Figure 1, the number of ED visits rose from 5 a.m. until reaching its highest level around noon. It stayed around peak volume until 6 p.m., and then decreased sharply—reaching its lowest volume just before 5 a.m. There may be many factors related to staffing, total number of patients in the ED, especially during the night shift, that contribute to the change over time. We further explored the relationship between admission hour and duration of ED visits by hospital characteristics. As presented in Figures 2 and 3, at both teaching and non-teaching hospitals, the mean duration of ED visits increased from 8 a.m. until noon, then decreased until midnight, at which time we observed spikes in mean duration of ED visits of 96 minutes at teaching hospitals, and 89 minutes at non-profit hospitals. In contrast, we did not observe a substantial increase in mean duration at for-profit or public hospitals. As shown in Figure 4, the mean duration of ED visits increased from 6 p.m. until midnight, at which time we observed a 41-minute spike in mean duration. The mean duration of ED visits at public hospitals was stable when compared to other hospital types. Figure 5 shows that there was a slight increase in mean duration of ED visits at public hospitals during the early morning and late night hours. As shown in Figures 2 and 3, the patterns of the variation of median and mean duration of ED visits throughout the day at teaching hospitals and non-profit hospitals were similar. However, at for-profit hospitals and public hospitals, the median duration was very stable at around 120 minutes throughout the day (Figures 4 and 5).

**Figure 6:**
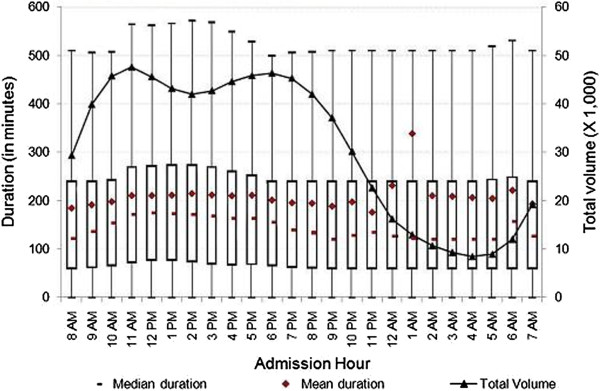
**Duration of treat-and-release visits at emergency departments on Mondays by hour.** Data includes all treat-and-release emergency visits during 2008 in Arizona, Massachusetts and Utah. Duration is measured in minutes as the difference between admission time and discharge time for each visit.

**Figure 7:**
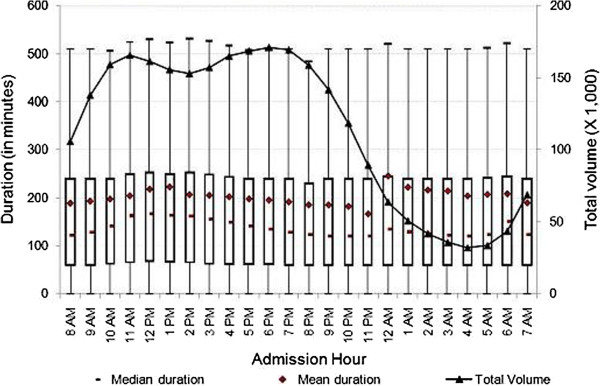
**Duration of treat-and-release visits at emergency departments on Non-Monday weekdays by hour.** Data includes all treat-and-release emergency visits during 2008 in Arizona, Massachusetts and Utah. Duration is measured in minutes as the difference between admission time and discharge time for each visit.

**Figure 8:**
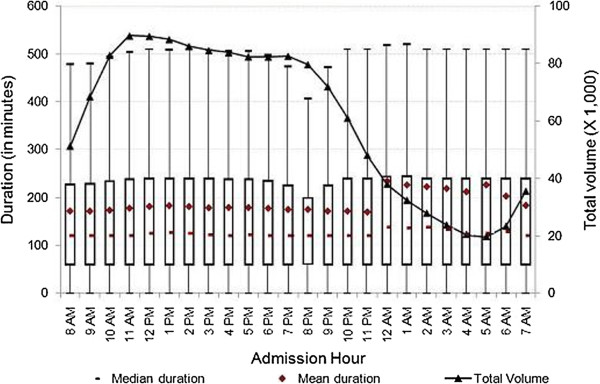
**Duration of treat-and-release visits at emergency departments on weekends by hour.** Data includes all treat-and-release emergency visits during 2008 in Arizona, Massachusetts and Utah. Duration is measured in minutes as the difference between admission time and discharge time for each visit.

There is growing concern among healthcare providers and policymakers about ED LOS on Mondays. We repeated the secondary data analyses to empirically show the differences, if any, between visits on Mondays and other weekdays or the weekend. As shown in Figures 6, 7, and 8, the mean duration of ED visits on the weekend were slightly shorter than that for visits on Mondays or other weekdays. For example, the mean duration of ED visits for patients arriving at 8 a.m. on Mondays, other weekdays, and weekends were about 184, 189, and 172 minutes, respectively. We found sizable difference in mean duration of ED visits between Mondays and other weekdays (over 90 minutes) only during the transition time from the evening of the day before to early morning hours (i.e., between midnight and 2 a.m.). While we observed a systematic increase in mean duration of ED visits during hours near midnight regardless of the admission day, we calculated that the increase was substantially larger on Mondays when compared to other weekdays or the weekend. This finding supports the hypothesis that our nation’s EDs may lack adequate resources to see patients on a typical Monday [14]. 

The mean, median and inter-quartiles (i.e., 25th and 75th quantiles) of duration across admission hours for the various characterizations reported in Figures 1, 2, 3, 4, 5, 6, 7, 8 showed very little variations. While the upper range was consistent at about 500 minutes for all visits (Figure 1), there were some variations for specific characterizations (i.e., Figure 2, teaching hospitals).

#### Patient characteristics

We analyzed patient demographics to explore potential explanations for the long duration of ED visits we observed (194.2 – 197.2 minutes). Table 1 displays the total number of T&R ED visits, mean duration of visits, and corresponding 95% confidence intervals for various patient characteristics. As shown in Table 1, the mean duration of visit increased with the age of the patient. We observed that the mean duration of ED visits for patients over 74 years of age was noticeably higher when compared to visits for patients younger than 15 years of age (237.5 versus 142.2 minutes). We also observed about 15 minutes longer mean duration of ED visits for female patients when compared to male patients.

**Table 1:** Mean and median duration, and total volume of treat-and-release visits at EDs by patient characteristics

	**Total Visits**	Mean Duration*	Median Duration*	**95% Confidence Interval for Mean**
All visit	4,955,590	195.7	130.2	(194.2 - 197.2)
Age				
Under 15	946,742	142.2	120.0	(141.6 - 142.7)
15 – 24	875,470	183.9	121.2	(182.7 - 185.1)
25 – 44	1,545,098	203.1	132.6	(199.0 - 207.2)
45 – 64	1,007,553	223.7	167.4	(220.0 - 227.4)
65 – 74	253,177	227.3	180.0	(222.9 - 231.6)
Over 74	327,421	237.5	180.0	(236.2 - 238.9)
Gender				
Male	2,305,226	187.4	120.0	(185.9 - 189.0)
Female	2,650,203	202.8	149.4	(200.4 - 205.3)
Race				
White	3,335,431	190.6	126.6	(189.8 - 191.3)
Black	354,549	212.0	154.2	(208.7 - 215.2)
Hispanic	914,958	202.4	146.4	(201.1 - 203.8)
Asian	73,124	203.8	153.0	(201.1 - 206.5)
Native	69,377	204.7	120.0	(193.3 - 216.1)
Other	83,967	193.8	141.0	(192.1 - 195.4)
Insurance Coverage				
Medicare	737,230	237.7	180.0	(236.0 - 239.4)
Medicaid	1,344,182	182.8	120.0	(181.7 - 183.9)
Private	1,990,780	192.8	130.2	(189.6 - 196.0)
Other insurance	239,412	169.4	120.0	(165.8 - 173.0)
Uninsured	532,653	191.8	120.0	(185.6 - 198.0)
CCS Disease Categories**				
[1 - 10]	262,850	184.3	120.0	(182.6 - 185.9)
[11 - 47]	72,107	286.8	237.0	(281.1 - 292.5)
[48 - 58]	507,389	269.4	192.6	(267.1 - 271.7)
[59 - 64]	59,067	327.3	240.0	(322.9 - 331.6)
[65 - 75]	712,236	284.0	180.0	(282.8 - 285.2)
[76 - 95]	767,383	202.1	154.8	(200.4 - 203.8)
[96 - 121]	795,116	256.4	180.0	(252.2 - 260.6)
[122 - 134]	929,207	185.3	126.0	(184.6 - 186.1)
[135 - 155]	545,083	228.2	180.0	(227.1 - 229.3)
[156 - 175]	454,092	241.2	182.4	(239.7 - 242.7)
[176 - 196]	123,116	237.0	187.2	(233.5 - 240.5)
[197 - 200]	245,968	160.0	120.0	(158.4 - 161.6)
[201 - 212]	642,445	198.1	136.2	(192.6 - 203.6)
[213 - 217]	15,454	221.0	180.0	(215.2 - 226.7)
[218 - 224]	11,601	140.5	120.0	(136.1 - 144.8)
[225 - 244]	1,449,389	159.6	120.0	(158.5 - 160.6)
[245 - 259]	1,370,770	228.7	180.0	(224.8 - 232.6)

Note: Data include all treat-and-release discharges at emergency departments during 2008 in Arizona, Massachusetts, and Utah.

* Duration is measured in minutes as the difference between admission and discharge time for each visit.

** Disease categories are based on Clinical Classification Software, which is a diagnosis and procedure categorization scheme based on the International Classification of Diseases, 9th Revision, Clinical Modification (ICD-9-CM).The HCUP SEDD do not differentiate between the primary diagnosis codes and other diagnosis codes. Therefore, we used all diagnosis codes reported for each visit when creating broader CCS disease categories. CCS disease codes: [1-10] infectious and parasitic diseases; [11-47] neoplasm; [48-58] endocr, nutri, metab, immun diseases; [59-64] diseases of blood, blood forming organs; [65-75] mental disorders; [76-95] diseases of nervous systems and sense organs; [96-121] diseases of circulatory systems; [122-134] diseases of respiratory systems; [135-155] diseases of digestive systems; [156-175] diseases of genitourinary systems; [176-196] complications of birth, pregnancy, puerperium; [197-200] diseases of skin and subcutaneous tissue; [201-212] diseases of musculoskel, connective tissue; [213-217] congenital anomalies; [218-224] perinatal conditions; [225-244] injury and poisoning; [245-259] other conditions. Further details are available at http://www.hcup-us.ahrq.gov/toolssoftware/ccs/ccs.jsp.

We also analyzed the mean duration of ED visits across race groups. As show in Table 1, the duration of ED visits for black/African American and Hispanic patients, respectively, was 11.2% and 6.2% longer than the duration of visits for non-Hispanic white patients. Our results support the findings of Herring et al. (2009) who found longer ED LOS for black/African American non-Hispanic patients (10.6% longer) and Hispanic patients (13.9% longer) when compared to non-Hispanic white patients.

Next, we analyzed the mean duration of ED visits by insurance coverage type. We found that Medicare patients’ visits had the longest mean duration (237.7 minutes), which could be due to higher severity of illness and presence of multiple diseases among these patients. Similarly, as shown in Table 1, the mean duration of ED visits for patients with Medicaid, private insurance, other insurance (e.g., TRICARE, worker’s compensation, health safety net, and other government payments or non-managed care plans) and no insurance coverage were 182.8, 192.8, 169.4, and 191.8 minutes, respectively. These results suggest that the difference in mean duration of ED visits between patients with any insurance coverage and uninsured patients is negligible. This result can also be interpreted as a positive sign that uninsured patients face limited barriers to healthcare access at emergency department settings.

Finally, we explored the potential relationship between the mean duration of visits and various disease groups as an assessment of severity of illness. As presented in Table 1, patients with diseases of blood and blood forming organs, neoplasm, and mental disorders experienced the longest mean duration of ED visits (327.3, 286.8, and 284.0 minutes, respectively). We observed the shortest ED stays among patients diagnosed with diseases of skin and subcutaneous tissue, injury and poisoning, and perinatal conditions (160.0, 159.6, and 140.5 minutes, respectively). These results highlight the impact of clinical severity of diseases on the mean duration of ED visits.

#### Hospital characteristics and area characteristics

Next, we analyzed hospital and area characteristics to explore other potential associations with longer ED visits. As shown in Table 2, hospitals with large bed size^g^ were associated with the longest duration of visits (222.2 minutes) when compared to hospitals with small bed size (172.4 minutes) or with medium bed size (166.5 minutes). Similarly, the mean duration of ED visits at urban hospitals was 26.8 minutes longer than those at their rural counterparts. Recognizing the differences in income levels across geographic regions, we compared the mean duration based on income distribution. We did not find significant differences in mean duration of ED visits between relatively richer or poorer counties. We also observed that the mean duration of visits at hospitals that are members of a hospital system was 6.7% shorter when compared to non-member hospitals. Similarly, the mean duration of visits at Level 1 trauma centers was 278.2 minutes and substantially longer than those at Level 2 or Level 3 trauma centers or non-trauma centers. One plausible explanation for this result is that Level 1 trauma centers provide the highest level of surgical care to seriously injured patients who may use more resources and whose treatments last longer. In contrast to visits at Level 1 trauma centers, the mean duration of visits at Level 2 and Level 3 trauma centers were shorter by more than 45 minutes compared to those at the non-trauma centers. When we looked more closely at the discharge positions of patients at non-trauma centers versus those at Level 2 and 3 trauma centers, we found that non-trauma centers have a relatively higher shares of patients transferred to short-term hospitals or other facilities. It might be plausible to assume that relatively higher shares of patients discharged to other facilities might be driving the difference since this discharge position is generally associated with longer duration of ED visits.

**Table 2:** Mean and median duration, and total volume of treat-and-release visits at EDs by hospital and area characteristics

	**Total Visits**	Mean Duration*	Median Duration*	**95% Confidence Interval for Mean**
All visits	4,955,590	195.7	130.2	(194.2 - 197.2)
Hospital State				
Arizona	1,782,239	215.6	180.0	(214.6 - 216.5)
Massachusetts	2,486,172	197.1	139.8	(194.2 - 200.1)
Utah	687,179	138.4	60.0	(137.4 - 139.5)
Hospital Location				
Rural	633,670	172.9	120.0	(163.2 - 182.6)
Urban	4,134,985	199.7	135.6	(198.7 - 200.7)
Teaching Status				
Teaching	1,427,968	243.8	180.0	(241.2 - 246.4)
Non-teaching	3,313,266	175.6	120.0	(173.6 - 177.5)
Ownership Status				
Public	304,453	180.0	120.0	(179.1 - 181.0)
Non-profit	3,516,831	202.5	148.2	(200.4 - 204.6)
For-profit	932,832	178.4	120.0	(177.6 - 179.3)
System Status				
Member	1,503,759	187.6	128.4	(185.5 - 189.8)
Non-member	3,264,896	200.1	130.8	(198.0 - 202.1)
Other Characteristics				
Non-trauma centers	3,704,109	187.8	121.2	(186.0 - 189.5)
Level 1 trauma centers	708,644	278.2	186.0	(273.0 - 283.4)
Level 2 trauma centers	285,894	140.6	115.2	(139.7 - 141.4)
Level 3 trauma centers	256,943	141.9	112.8	(139.9 - 144.0)
Hospital Bed Size				
Small	1,013,957	172.4	121.2	(171.1 - 173.6)
Medium	1,335,735	166.5	120.0	(164.0 - 169.0)
Large	2,418,963	222.2	177.6	(219.5 - 224.9)
Median Household Income**				
$1 - $38,999	1,046,901	197.6	129.0	(196.2 - 198.9)
$39,000 - $48,999	1,229,219	198.2	127.2	(194.5 - 201.9)
$49,000 - $63,999	1,178,364	193.8	125.4	(189.1 - 198.4)
$64,000 and over	1,265,601	195.6	139.8	(194.3 - 197.0)

Note: Data include all treat-and-release discharges at emergency departments during 2008 in Arizona, Massachusetts, and Utah.

* Duration is measured in minutes as the difference between admission and discharge time for each visit.

** This is a quartile classification of the estimated median household income of residents in the patient's ZIP Code. Further details are available at http://www.hcup-us.ahrq.gov/db/vars/zipinc_qrtl/nisnote.jsp.

Table 2 also shows that the mean duration of visits at teaching hospitals was substantially higher than at non-teaching hospitals (243.8 versus 175.6 minutes). The mean duration of visits at public, non-profit, and for-profit hospitals was 180.0, 202.5, and 178.4 minutes, respectively, showing significant differences between for-profit and non-profit hospitals (where duration was 13.5% longer). One plausible reason for the difference could be the different financial incentives for for-profit and non-profit hospitals. We further analyzed the mean duration of visits throughout the day to uncover any significant differences. Figure 3 shows that the mean duration at non-profit hospitals was substantially higher for the majority of the day when compared to for-profit hospitals, except between 8 p.m. and 1 a.m. During the late evening period, non-profit hospitals showed lower mean duration when compared to for-profit hospitals. For example, the mean duration of ED visits from 10 p.m. to 12 a.m. was about 70 minutes shorter at non-profit hospitals when compared to their for-profit hospitals.

Finally, we analyzed patients’ discharge disposition from EDs by hospital and area characteristics to further explore other potential associations with longer ED visits. As shown in Table 3, the mean duration of ED visits for patients discharged to home health care was substantially higher when compared to patients discharged elsewhere. The mean duration of visits for patients transferred to home health care and other long-term care facilities were about 871 minutes and 507 minutes respectively. The mean duration of ED visits for patients discharged home and patients discharged against medical advice were about 187 and 209 minutes, respectively. As presented in Table 3, the mean duration for patients visiting EDs at urban hospitals were substantially higher when compared to rural hospitals regardless of patients’ discharge disposition. Similarly, mean duration of visits at teaching hospitals relative to non-teaching hospitals and at non-profit hospitals relative to for-profit were considerably longer for patients transferred to short-term hospitals or other facilities. The mean duration of ED visits was also higher in Level 1 trauma centers when compared to non-trauma, Level 2, and Level 3 trauma centers across patients’ discharge status, except when the patient died in the hospital. Patients visiting EDs of hospitals with large bed size experienced longer duration regardless of their discharge status when compared to hospitals with small or medium bed sizes. Finally, the mean duration of ED visits at hospitals that were members of a hospital system was slightly higher when compared to hospitals that were not members of hospital systems.

**Table 3:** Mean and median duration of treat-and-release visits at EDs by disposition of the patient at discharge across hospital and area characteristics

	**Routine**		**Transfer to short term hospitals**		Transfer to other facilities*		Home health care**		**Against medical care**		**Died**	
	**Mean**	**Median**	**Mean**	**Median**	**Mean**	**Median**	**Mean**	**Median**	**Mean**	**Median**	**Mean**	**Median**
All visits	186.9	126.0	288.7	240.0	507.2	304.2	871.0	420.0	209.0	145.8	156.9	60.0
Hospital State												
Arizona	207.8	180.0	316.3	240.0	630.4	420.0	650.8	360.0	220.2	120.0	144.3	60.0
Massachusetts	186.0	135.6	.	.	488.6	297.6	.	.	206.9	169.2	170.2	68.4
Utah	136.5	60.0	157.8	120.0	287.6	120.0	1,315.2	1,170.0	121.6	.	138.6	60.0
Hospital Location												
Rural	164.8	120.0	271.5	240.0	414.4	261.0	.	.	179.2	120.0	126.8	3.6
Urban	190.5	130.8	292.2	240.0	525.6	318.0	904.9	420.0	212.7	154.2	163.5	60.0
Teaching Status												
Teaching	215.5	171.6	317.9	240.0	595.0	360.0	826.9	420.0	235.7	180.0	145.3	60.0
Non-teaching	158.4	120.0	261.7	240.0	424.2	171.0	972.6	360.0	163.7	120.0	173.6	68.4
Ownership Status												
Public	171.3	120.0	312.0	240.0	458.4	268.2	.	.	169.4	120.0	111.2	60.0
Non-profit	192.3	139.2	297.3	240.0	530.3	324.0	658.6	300.0	233.6	180.0	168.0	66.9
For-profit	173.9	120.0	256.4	180.0	428.1	280.2	1,298.4	1140.0	138.9	60.0	140.7	60.0
System Status												
Member	191.7	124.8	280.3	240.0	515.9	312.0	911.4	420.0	224.0	133.2	138.2	60.0
Non-member	177.2	126.6	303.0	240.0	502.1	300.6	.	.	184.9	157.8	205.9	81.3
Other Characteristics												
Non-trauma centers	179.8	120.0	286.7	240.0	473.4	298.8	910.7	420.0	195.4	120.0	169.2	78.6
Level 1 trauma centers	264.9	183.0	376.0	300.0	725.8	459.6	.	.	278.1	183.8	142.2	54.3
Level 2 trauma centers	132.8	112.8	.	.	425.7	285.6	.	.	152.4	121.2	108.3	34.2
Level 3 trauma centers	134.2	109.8	.	.	397.1	279.4	.	.	173.8	152.1	117.6	59.4
Hospital Bed Size												
Small	163.4	120.0	234.5	180.0	425.6	268.2	462.8	180.0	175.5	127.2	139.8	105.0
Medium	159.4	120.0	262.7	240.0	438.0	272.4	741.3	360.0	157.1	120.0	197.9	60.0
Large	212.1	172.8	329.3	300.0	600.4	367.8	1,068.4	540.0	235.3	180.0	143.4	60.0
Median Household												
$1 - $38,999	190.1	124.8	282.0	240.0	546.5	360.0	859.3	480.0	200.6	121.8	125.7	60.0
$39,000 - $48,999	190.2	123.0	296.5	240.0	530.3	318.3	1,052.3	480.0	218.2	159.9	133.6	60.0
$49,000 - $63,999	184.9	121.8	287.0	240.0	482.8	295.2	936.9	480.0	207.2	144.0	191.9	70.2
$64,000 and over	184.9	134.4	292.2	240.0	501.7	301.2	622.6	240.0	203.6	165.6	167.4	60.0

Note: Data include all treat-and-release discharges at emergency departments during 2008 in Arizona, Massachusetts, and Utah. Duration is measured in minutes as the difference between admission and discharge time for each visit. Statistics are not reported whenever there are fewer than 100 observations in a particular cell.

* Other facilities include skilled nursing facility, intermediate care facility, and other types of similar facilities.

** Home health care facilities also include IV providers and hospice home care facilities. Detailed description for each discharge.

### Regression results

Table 4 presents regression results that convey the impact of admission day of the week, patient demographics, and hospital characteristics on duration of patients’ visits to EDs. All results are highly statistically significant for all variables across all models except hospital characteristics estimated under the multilevel model. Average duration of visits on Mondays is at least 4 percent and 9 percent more than the average duration of visits on non-Monday workdays and on weekends, respectively. The results also show that average duration of ED visits for older patients or female patients is generally longer when compared to younger patients or male patients. Non-white patients generally experience longer duration of ED visits when compared to white patients. When compared to patients with other primary payers, Medicare patients are generally associated with longer duration of ED visits, and uninsured patients or patients who pay out-of-pocket are generally associated with shorter duration of ED visits.

**Table 4:** Estimated Effects of Admission Time, Patient and Hospital Characteristics on Log Duration of Emergency Department Visits

	**Linear Regression Models**						**Multilevel Model**	
	**Model 1**		**Model 2**		**Model 3**		**Model 4**	
	**Parameters**	**Std. Errors**	**Parameters**	**Std. Errors**	**Parameters**	**Std. Errors**	**Parameters**	**Std. Errors**
Admission Day ^1^								
Visit was on Monday	0.045***	(0.002)	0.044***	(0.002)	0.045***	(0.002)	0.041***	(0.002)
Visit was on weekend	−0.057***	(0.002)	−0.059***	(0.002)	−0.056***	(0.001)	−0.055***	(0.001)
Patient Demographics Characteristics								
Age	0.014***	(0.000)	0.015***	(0.000)	0.013***	(0.000)	0.013***	(0.000)
Age-squared	0.001***	(0.000)	0.001***	(0.000)	0.001***	(0.000)	0.001***	(0.000)
Female	0.049***	(0.002)	0.046***	(0.002)	0.035***	(0.001)	0.039***	(0.001)
White	0.077***	(0.004)	−0.031***	(0.004)	0.146***	(0.003)	0.137***	(0.003)
Black	0.107***	(0.005)	0.007	(0.005)	0.179***	(0.003)	0.165***	(0.003)
Hispanic	0.115***	(0.004)	0.013***	(0.004)	0.131***	(0.003)	0.122***	(0.003)
Asian	0.267***	(0.007)	0.162***	(0.007)	0.231***	(0.005)	0.222***	(0.005)
Medicare	0.103***	(0.004)	0.108***	(0.004)	0.149***	(0.003)	0.152***	(0.003)
Medicaid	−0.143***	(0.003)	−0.141***	(0.003)	0.094***	(0.002)	0.096***	(0.002)
Private	0.089***	(0.003)	0.101***	(0.003)	0.116***	(0.002)	0.119***	(0.002)
Self-paid	−0.149***	(0.004)	−0.146***	(0.004)	−0.037***	(0.003)	−0.036***	(0.003)
Hospital Characteristics								
Teaching hospitals	0.146***	(0.002)	0.144***	(0.002)	.	.	0.125	(0.282)
For-profit hospitals	−0.596***	(0.002)	−0.599***	(0.002)	.	.	−0.318	(0.313)
Trauma Level 1 hospitals	0.418***	(0.003)	0.411***	(0.003)	.	.	0.557	(0.453)
Trauma Level 2 hospitals	−0.968***	(0.004)	−0.959***	(0.004)	.	.	−0.352	(0.634)
Trauma Level 3 hospitals	−0.731***	(0.004)	−0.728***	(0.004)	.	.	−0.693	(0.587)
Medium size hospitals^2^	−0.361***	(0.002)	−0.354***	(0.002)	.	.	−0.168	(0.308)
Large hospitals	−0.119***	(0.002)	−0.112***	(0.002)	.	.	0.041	(0.312)
Member hospitals	−0.312***	(0.002)	−0.307***	(0.002)	.	.	−0.322	(0.263)
Urban hospitals	0.095***	(0.003)	0.091***	(0.003)	.	.	0.207	(0.320)
Control for CCS codes^3^	Yes		Yes		Yes		Yes	
Control for admission hour^4^	No		Yes		Yes		No	
Control for hospital^5^	No		No		Yes			
Total number of visits	4,768,411		4,761,855		4,761,855		4,718,553	
R-square	0.096		0.097		0.542			
Inra-class correlation	.		.				0.565	

Note: Data include all treat-and-release discharges at emergency departments during 2008 in Arizona, Massachusetts, and Utah. Duration is measured in minutes as the difference between admission and discharge time for each visit.

1 The control group was the average of non-Monday workdays.

2 Further details about hospital bed sizes are available at http://www.hcup-us.ahrq.gov/db/vars/hosp_bedsize/nisnote.jsp.

3 Disease categories are based on Clinical Classification Software, which is a diagnosis and procedure categorization scheme based on the International Classification of Diseases, 9th Revision, Clinical Modification (ICD-9-CM).The HCUP SEDD do not differentiate between the primary diagnosis codes and other diagnosis codes. Therefore, we used all diagnosis codes reported for each visit when creating broader CCS disease categories.

4 We created dummy variables for each hour of the day.

5 We created dummy variables for each hospital.

*** P<0.001; ** P<0.05; * P<0.10.

The regression results presented in Table 4 show that patients visiting teaching hospitals and Level 1 trauma centers generally experience longer duration of ED visits. Average duration of patient’s visits to Level 2 and Level 3 trauma centers are generally shorter when compared to the duration of ED visits at non-trauma hospital centers. Patients visiting urban hospitals experience longer duration of ED visits when compared to patients visiting rural hospitals. Similarly, the average duration of ED visits to hospitals with large or medium bed size is shorter than the average duration of ED visits to hospitals with small bed size. Table 4 also provides crucial information about the source of variation in duration of ED visits. The intra-class correlation coefficient obtained through multilevel regression analysis indicates that about 56 percent of variations in duration of patients’ visits to EDs are due to variation within patients clustered by hospitals. Alternatively, hospitals are accountable for less than 45 percent of total variations in duration of ED visits.

### Limitations

As mentioned earlier, measures of timeliness of care in the ED that have been advanced in the literature are not available in HCUP data. Therefore, we computed the duration for each visit by taking the difference between admission and discharge times, which is the total time patients were waiting in the ED plus their treatment and discharge times.

The HCUP SEDD data is based on ED encounters as the unit of analysis, so a given patient may have many visits. As a consequence, the summary information reported under patient characteristics might overestimate or underestimate demographics for individual patients. Finally, this study does not address the impact of financial incentives and other confounding factors across hospitals types on duration of ED visits.

Our analysis is confined to the T&R ED data presented in the HCUP SEDD from only three states: Arizona, Massachusetts, and Utah. Relatively small sample sizes may contribute to some of our findings, such as observing a skew in duration around Monday midnight. ED encounters that result in subsequent admission to the same hospital are not included in the analysis. Patients that are admitted, and perhaps boarded, might have different experiences than those presented in our results. There can also be considerable variations at the facility-level in the rate at which patients are admitted from the ED. Therefore, the EDs contained in this analysis may have considerably different mixes in the number of patients that they treat and release and those that they admit.

## Conclusions

Our results show that the mean duration for a T&R ED visit was slightly above 3 hours and it varied considerably by admission hour and day of the week, patient volume, patient characteristics, hospital characteristics and area characteristics. When documenting the mean duration, we uncovered a significant spike in mean duration of ED visits at around midnight, occurring mostly on Monday nights at for-profit hospitals. Based on patient demographics and hospital characteristics, we identified several important factors that are associated with increased ED stays. We identified a direct relationship between increased duration of T&R ED visits and patient age, race, gender, and severity of illness; and hospital ownership type and location. Elderly patients, patients with mental disorders or neoplasm, non-white patients, and female patients experienced longer ED stays than did other patients. Consistent with existing literature, our results suggest that, in the aggregate, lack of health insurance did not have a significant direct association with longer mean duration of ED visits. The mean duration of ED visits was substantially longer at non-profit hospitals when compared to for-profit hospitals, and at Level 1 trauma centers when compared to other trauma centers or non-trauma centers. We also show that the mean duration of ED visits for patients discharged to home health care or other long term care facilities was substantially higher when compared to patients discharged home or elsewhere. Our findings may also inform public and private policymakers on a broad range of issues including, but not limited to, Monday volume, impact of hospital bed size and hospital status on the mean duration of T&R ED visits, and differences in duration by race.

Some of the results are consistent with the literature’s characterization of care provided in the ED and are expected. Level I trauma centers, for example, have comprehensive resources and are able to care for the most severely injured patients. They also provide leadership in education and research. Therefore, it is not surprising that they have the longest duration for T&R patients. Other findings are not as easy to interpret. We found earlier that a larger share of patients transferred to short-term hospitals or other facilities could be one of the contributing factors for longer duration of visits at non-trauma hospitals when compared to Level 2 or Level 3 trauma centers. However, it is still not clear why non-trauma hospitals should have a longer duration than Level 2 or Level 3 trauma centers.

Many of these findings are worthy of further exploration. For example, we believe that since elderly patients frequently present to the ED with multiple complications, they require more ED resources during their visits, which causes them to have a longer duration of visit. Similarly, one plausible explanation for midnight spike in duration on Mondays might be that healthcare personnel change shifts at this time and/or a reduction in other resources between 11 p.m. and midnight. Another plausible explanation might be that healthcare personnel might experience decrease in their labor productivity towards ends of their shifts. Some researchers may claim that our multilevel model estimates produced higher intra-class correlations since the higher the intra-class correlation, the less unique the information provided by each additional patient. Nonetheless, our goal is to show the source of variation between hospitals and patients. Further research using more clustering with fewer cases per cluster is warranted. We also believe that our findings may provide unique opportunities for quality improvements within hospital emergency departments, as we presented sizable variation in duration of T&R ED visits across a wide range of patient and hospital characteristics.

## Endnotes

^a^Further details about these data files are available at http://www.cdc.gov/nchs/ahcd.htm

^b^Further details about HCUP databases are available at http://www.hcup-us.ahrq.gov/

^c^Further details are available at http://www.hcup-us.ahrq.gov/tech_assist/centdist.jsp

^d^As part of the HCUP Project, AHRQ negotiates with data organizations that maintain statewide data systems to acquire hospital-based data, process those data into research databases, and subsequently release a subset of those data to the public with a signed data use agreement. Some data elements are considered too sensitive by these data organizations for general release to the public. However, under the terms of their agreements with AHRQ, some AHRQ staff may use these more sensitive data for analysis. In this study, the Arizona Department of Health Services, the Massachusetts Division of Health Care Finance and Policy, and the Utah Department of Health granted permission for the data elements, admission hour and discharge hour, to be used internally by AHRQ.

^e^ARF provide county level data. Further details are available at http://arf.hrsa.gov/

^f^We focus mainly on the mean value of duration in our analysis. However, we have provided both mean and median values for each measure separately throughout all tables and figures to set the stage for further research and to provide additional detail to key policymakers and curious researchers.

^g^Further details about hospital bed sizes are available at http://www.hcup-us.ahrq.gov/db/vars/hosp_bedsize/nisnote.jsp

## Competing interests

The authors declare no potential competing interests with respect to the authorship and/or publication of this article. The views expressed herein are those of the authors. No official endorsement by any agency of the federal or state governments is intended or should be inferred.

## Authors’ contributions

ZK and HSW conceived the study. ZK, HSW and RLM provided policy advice on the findings of this paper; ZK provided the statistical analysis plan and analyzed the data. ZK has been the primary author of the manuscript while all other authors contributed to the writing of the manuscript and read and approved the final manuscript.

## Pre-publication history

The pre-publication history for this paper can be accessed here:

http://www.biomedcentral.com/1471-227X/12/15/prepub
